# Notes and News

**Published:** 1895-08-31

**Authors:** 


### NOTES AND NEWS.

(Collected and Communicated.)

It may be of interest for those who contemplate attending the International Congress of Otology to know that Messrs. Cook and Son are making special travelling arrangements for the occasion, all particulars of which they have published, and which can be obtained on application at their offices.

The War Office have instructed Messrs. Paterson and Cooper to proceed with the erection at Netley Hospital of the Hermite sanitation plant, the cost of which has been provided for in this year's Parliamentary estimates. The Corporation of Cape Town have also ordered a plant for producing electrolised sea water with a view to the Hermite process being applied to the sanitation of Cape Town.

The right of trial by jury is one of the great bulwarks of our freedom, and one which we may not lightly cast aside. It cannot be denied, however, that the system inflicts very great hardships on individuals, both in regard to the frequency with which they are called to serve, and the inordinate length of time over which many trials are now made to last. It behoves us, then, not unnecessarily to extend the system lest it should fall to pieces under the strain. At a recent meeting of the Personal Rights Association it was maintained by the reader of a paper upon the lunacy laws that "no one ought to be declared of unsound mind except on the verdict of a jury upon sworn evidence taken in open court." This is all very well, but how about the 18,000 to 20,000 juries which would have to be called together for the purpose every year? If the magistrates do their duty there are ample provisions in the lunacy laws for the protection of the public, and the more our asylums become institutions for the treatment rather than the mere detention of the insane, the more desirable is it that the admission of patients who it is hoped may shortly come out cured may be attended with no more publicity than is absolutely required for the prevention of abuse.

The Memorial Cottage Hospital at Crewe was opened on August 14th by the Earl of Crewe. The building contains three wards for six beds each, and a smaller ward for two beds, with an operation-room, matron's and nurses' rooms, and the usual offices. The cost has been about £3,000.

The Westminster Hospital has been closed for necessary repairs, and especially for the relaying of the drains, which are very old. A strenuous effort is being made to increase the maintenance fund, an effort which certainly ought to be successful, for while on the one hand the hospital is doing good work very economically among people drawn from a very poor district in the immediate neighbourhood, on the other hand there is also in the near neighbourhood a large population of wealthy people living in the flats which are so rapidly growing up around Victoria Street, and it ought to be possible to collect from them a large amount annually for the help of their poorer neighbours. If even small contributions were given regularly by the large number of well-to-do residents in the district, the Westminster Hospital would no longer want for funds.

The addresses delivered at the opening of the medical schools mark what is to some the commencement of their career, to others only the recurrence of a time of work after their well-earned holidays. But for all they are the signal for a fresh start in intellectual activity, and however much some of the less thoughtful may jeer at "introductories," these addresses ought to be, and in many cases are, of great service not only to those entering on their medical studies, but in stimulating to higher efforts those already engaged in the daily practice of their profession. At St. Thomas's Hospital the introductory address will be given by Sir Edwin Arnold, at Middlesex by Dr. W. Julius Mickle, at St. Mary's by Mr. A. P. Laurie, at Westminster by Dr. Monckton Copeman, at St. George's by Mr. George D. Pollock, at University College by Professor J. Rose Bradford, and at Guy's by Mr. George De'Ath. Mr. Hutchinson will address the students at University College, Liverpool, and Mr. Victor Horsley those at the Sheffield School of Medicine.

The reported prevalence of ophthalmia among the children under the care of the Chester Guardians draws attention to the sad lack on the part of the Local Government Board either of the power or of the will to compel the Guardians to act in accordance with such knowledge as we possess regarding the causation of this disease. It is a satire upon the whole system of poor law administration that knowledge laboriously gathered by one set of Guardians at the expense of much money on the part of the ratepayers, and, it is to be feared, of much blindness and other suffering on the part of the children, should be ignored by other Guardians till they, their ratepayers, and their children in turn, have themselves to go through the same mill of sad experience. The causes of ophthalmia have been carefully worked out, and first among them stands the massing together of numbers of children with deficient air space and ventilation, and if, in view of the knowledge we now possess, it is astonishing to find guardians allowing the continuance of such conditions, it is still more astonishing and disheartening to find so powerful a body as the Local Government Board, with the highest expert advice at its disposal, exercising its influence and authority to such small effect that an immediate check is not put on such proceedings.

